# Conservative treatment followed by chemotherapy with doxorubicin and ifosfamide for cervical sarcoma botryoides in young females

**DOI:** 10.1038/sj.bjc.6690370

**Published:** 1999-05-01

**Authors:** G Zanetta, S M Rota, A Lissoni, S Chiari, G Bratina, C Mangioni

**Affiliations:** Departments of Obstetrics and Gynaecology, andSurgical Pathology, San Gerardo Hospital of Monza, Third Branch of the University of Milan, Via Solferino 16, 20052 Monza, Italy

**Keywords:** sarcoma botryoides, embryonal rhabdomyosarcoma, cervix, chemotherapy, conservative surgery

## Abstract

Sarcoma botryoides of the cervix is an extremely rare tumour and seems to be associated with a better prognosis than its vaginal counterpart. Recent studies have suggested that it is possible to limit surgery to local excision in stage I cases. We report three cases of young subjects treated successfully with polypectomy or diathermy loop excision followed by adjuvant chemotherapy. One patient had a local recurrence which was treated with further local excision. All subjects remain alive without evidence of recurrence and with normal menstrual function 36, 38 and 38 months following initial diagnosis. A conservative surgical approach to early cervical sarcoma botryoides is possible. The efficacy of adjuvant chemotherapy and the regimen of choice still need to be investigated. © 1999 Cancer Research Campaign


*British Journal of Cancer* (1999) **80**(3/4), 403–406
© 1999 Cancer Research Campaign
Article no. bjoc.1998.0370

### Conservative treatment followed by chemotherapy with doxorubicin and ifosfamide for cervical sarcoma botryoides in young females

**G Zanetta[1], SM Rota[1], A Lissoni[1], S Chiari[1], G Bratina[2] and C Mangioni[1]**

Departments of [1]Obstetrics and Gynaecology and [2]Surgical Pathology, San Gerardo Hospital of Monza, Third Branch of the University of Milan, Via Solferino 16, 20052 Monza, Italy

**Summary** Sarcoma botryoides of the cervix is an extremely rare tumour and seems to be associated with a better prognosis than its vaginal counterpart. Recent studies have suggested that it is possible to limit surgery to local excision in stage I cases. We report three cases of young subjects treated successfully with polypectomy or diathermy loop excision followed by adjuvant chemotherapy. One patient had a local recurrence which was treated with further local excision. All subjects remain alive without evidence of recurrence and with normal menstrual function 36, 38 and 38 months following initial diagnosis. A conservative surgical approach to early cervical sarcoma botryoides is possible. The efficacy of adjuvant chemotherapy and the regimen of choice still need to be investigated.

**Keywords:** sarcoma botryoides; embryonal rhabdomyosarcoma; cervix; chemotherapy; conservative surgery

Sarcoma botryoides, a variant of embryonal rhabdomyosarcoma, is an extremely rare tumour and is usually diagnosed in childhood. The Intergroup Rhabdomyosarcoma Study (IRS) reported that rhabdomyosarcomas usually arise from the vagina in infancy and in early childhood, whereas uterine rhabdomyosarcomas are more often seen during adolescence (Hays et al, 1985).

The treatment of this tumour has changed dramatically over the decades. In 1959, Daniel et al noted the frequent multicentric origin of this tumour (Daniel et al, 1959). In the late 1960s some authors (Rutledge and Sullivan, 1967) stated that pelvic exenteration was the treatment of choice for this tumour; however, a comprehensive review on the results of pelvic exenteration showed that the results were unsatisfactory, particularly when the tumour extended beyond the vagina (Hilgers, 1975).

During the 1970s combined treatment modalities including chemotherapy and/or irradiation with limited surgery provided better control of this disease and considerable improvement in survival (Holton et al, 1973; Ghavimi et al, 1975; Ortega, 1979). Cervical sarcoma botryoides has been associated with a more favourable outcome than other genital rhabdomyosarcomas and a more conservative approach has been advised under certain circumstances (Montag et al, 1986; Brand et al, 1987; Daya and Scully, 1988; Gordon and Montag, 1990; Lin et al, 1995).

This paper describes our recent experience with multiagent chemotherapy and limited surgery in the treatment of early cervical rhabdomyosarcoma in three young subjects.

*Received 19 May 1998*
*Revised 11 August 1998*
*Accepted 10 November 1998*

*Correspondence to:* G Zanetta

### CLINICAL HISTORIES

### Case 1

A 22-year-old woman, gravida 0, presented in December 1994 complaining of genital bleeding. Her previous gynaecological history was uneventful. On speculum examination a large cervical polyp, measuring $4 \times 4 \times 3$ cm, was seen and removed. Microscopic examination revealed a botryoid sarcoma with more than 30 mitoses per high-power field but, due to mechanical distorsion of the polyp at removal, it was not possible to define without doubts whether the margins were microscopically involved by tumour (Figures 1 and 2).

Lymphangiography, intravenous pyelogram, magnetic resonance of the pelvis and abdomen, transvaginal ultrasound, colposcopy and hysteroscopy were all negative. Endometrial and endocervical curettages and cervical biopsies in the area of the excised tumour were negative. She was therefore classified as stage IA according to the criteria of the IRS.

This patient received adjuvant chemotherapy with doxorubicin 25 mg $m^{-2}$ daily on days 1, 2 and 3 and ifosfamide 2 g $m^{-2}$ daily on days 1, 2 and 3. Doxorubicin was given as bolus, whereas ifosfamide was administered as a 24-h infusion. The treatment was repeated every 3 weeks for three courses, without delay. Myelotoxicity (WHO grade 3), alopecia (grade 3) and mucositis (grade 1) were the most relevant side-effects.

Follow-up abdominal and transvaginal ultrasound, hysteroscopy and colposcopy, revealed no evidence of residual disease. Random biopsies of the cervix were all negative. Her first menstrual period occurred 4 months after the end of the treatment. She was monitored closely, with colposcopy, hysteroscopy and ultrasound every 3 months. Nine months later, colposcopy revealed a small cervical polyp of approximately 1 cm in diameter. This was removed by

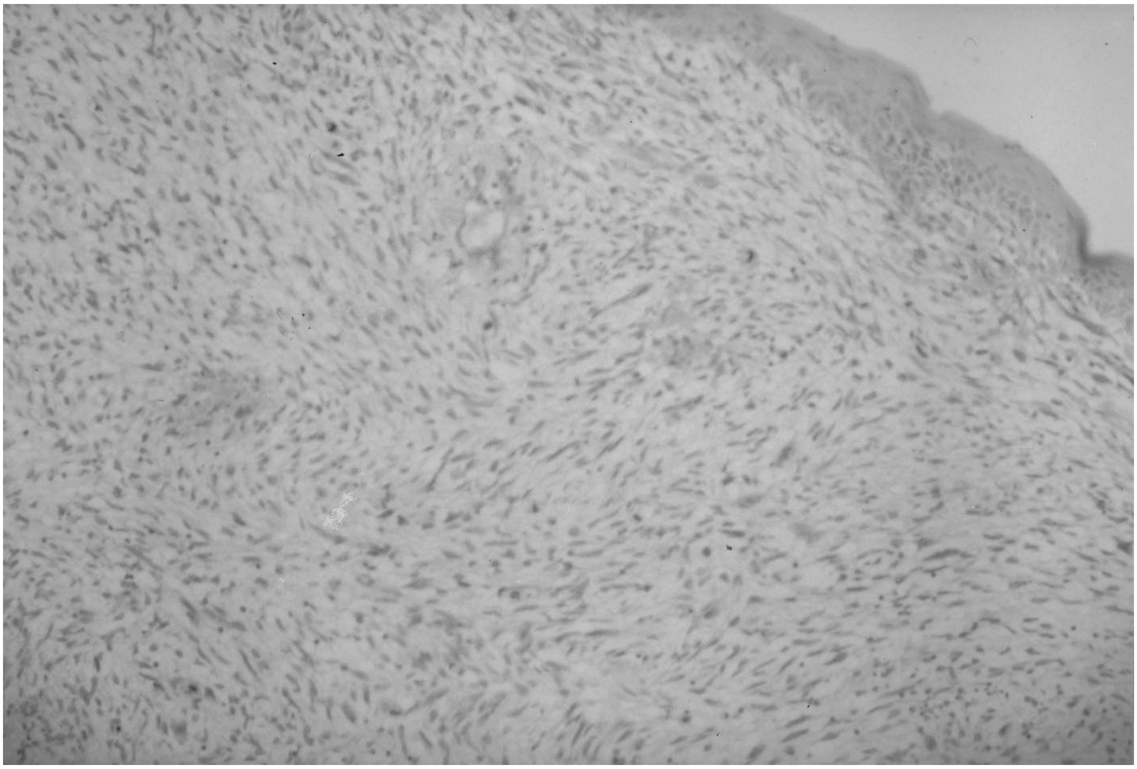

**Figure 1** Low-power photomicrograph shows a cervical polyp with spindle cells (H&E stain, magnification 25×)

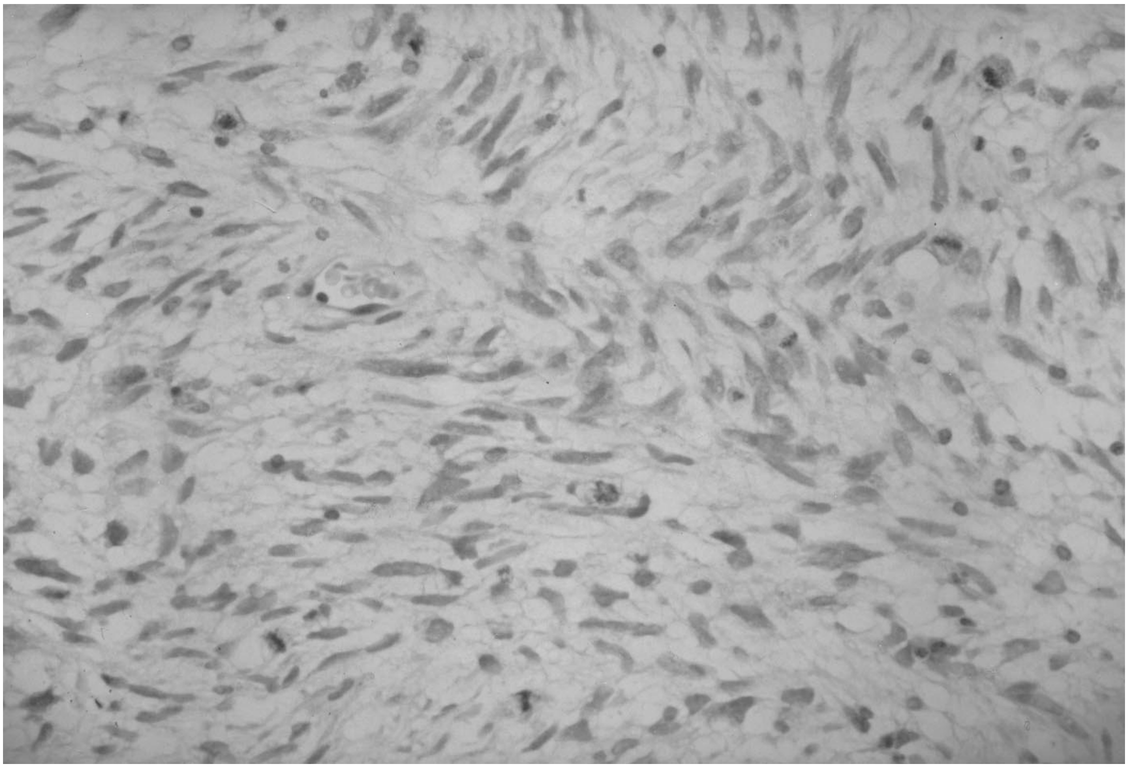

**Figure 2** High-power magnification shows spindle cells with mitoses (H&E stain; magnification 250×)

 *© Cancer Research Campaign 1999*

diathermic loop. Microscopic examination revealed recurrence of sarcoma botryoides with few mitoses. The margins were all negative and she was followed without further treatment. She continued to show no evidence of local or distant recurrence 38 months after the initial diagnosis.

### Case 2

A 25-year-old, gravida 0, presented in February 1995 to a primary care hospital, complaining of vaginal bleeding which had lasted for 3 weeks. A cervical polyp was discovered and removed by torsion. Histopathologic analysis was considered benign and the patient did not receive additional therapy. In August 1995 she noted again abnormal vaginal bleeding. On speculum examination a cervical polyp was noticed and a polypectomy was again performed.

Microscopic examination revealed a rhabdomyosarcoma with few mitoses and she was referred to our department of gynaecological oncology. Review of the specimen removed in February detected the presence of sarcoma botryoides. Magnetic resonance imaging of the abdomen and the pelvis detected no metastatic lesions. Colposcopy did not reveal any additional lesion but hysteroscopy detected a small polyp arising from the inner third of the cervical canal. The polyp was removed by means of endocervical curettage and the microscopic examination confirmed the persistence of sarcoma botryoides with involved margins. The lesion was located too deeply in the endocervical canal to allow a conization and no visible tumour remained after the curettage, therefore we decided to treat the patient and to repeat the hysteroscopy after each course of chemotherapy in order to detect as soon as possible a local persistence. We decided to leave the possibility of a cervicectomy or a hysterectomy only in case of persistence under chemotherapy or in case of recurrence. She received four courses of chemotherapy with the same regimen described above. She experienced WHO grade 3 myelotoxicity, grade 3 vomiting and grade 3 alopecia but did not require any delay and completed the treatment by December 1995. Her first menstrual period occurred in April 1996. She remained disease-free 24 months following chemotherapy and 36 months following initial diagnosis.

### Case 3

A 20-year-old, gravida 0, underwent gynaecological examination in March 1995, due to persistent irregular bleeding. Speculum examination revealed a large polypoid lesion which extended almost to the vaginal introitus. The polyp arose from the cervix by a thin pedicle and was completely excised. Pathologic analysis revealed sarcoma botryoides with more than 30 mitoses per high-power field.

She was referred to our division of gynaecological oncology where she underwent pelvic and abdominal computerized tomography (CT) scan, hysteroscopy, colposcopy and transvaginal ultrasound without evidence of residual disease. Three courses of chemotherapy, according to the regimen already described, were administered without delay. The most relevant side-effects were myelotoxicity grade 3, vomiting grade 2 and alopecia grade 3. Normal menstrual periods were recorded during the treatment and afterwards. She continued to show no evidence of local or distant recurrence 38 months following the initial diagnosis.

### DISCUSSION

The prognosis of patients with cervical sarcoma botryoides is rather favourable when compared to other genital rhabdomyosarcomas, particularly when the tumour arises in a single polypoid lesion and when the polyp is completely removed or resected (Perrone et al, 1990; Zeisler et al, 1998). The surgical aggressiveness for the management of this tumour has progressively decreased from pelvic exenteration (often used until the 1970s) to abdominal hysterectomy. Some authors have also proposed a more conservative approach in recent years for cases with no macroscopic residual tumour after excision of the primary lesion (Gordon and Montag, 1990; Lin et al, 1995).

Due to the lack of data on large populations about the outcome of patients managed conservatively, we decided to treat these patients with doxorubicin and ifosfamide after the excision of the polyp. Therefore all three subjects received chemotherapy in the absence of obvious residual tumour. Two of them had no recurrence but the third subject had detection of residual tumour 9 months after the end of chemotherapy. In this case, repeat local excision of the tumour was considered sufficient treatment because no deep invasion of the cervical stroma was documented and the patient remains disease-free.

This chemotherapy regimen was chosen because the combination of doxorubicin and ifosfamide yielded fairly satisfactory results in advanced or recurrent genital sarcomas in a previous multicentre study (Leyvraz et al, 1995). Our results do not allow any definitive conclusion about the effectiveness of this regimen in sterilizing minimal amounts of residual tumour. The use of different regimens, more prolonged administration or higher doses remains a matter of discussion. Some authors (Hays et al, 1985) have noticed that doxorubicin did not improve the outcome when added to pulse VAC (vincristine, dactinomycin, cyclophosphamide) for patients with macroscopic disease (Group III and IV). However, they also noticed that doxorubicin was given at a relatively low-dose intensity.

Pelvic irradiation could be proposed as an alternative to adjuvant chemotherapy. However, this treatment modality would interfere with the ovarian function of these young subjects, making conservative management meaningless. Although the follow-up is still relatively short, all our patients maintain a normal menstrual function. Their possible future fertility remains to be assessed.

The successful treatment of a shallow local recurrence with simple resection raises the question whether close observation without adjuvant treatment might be considered in those cases where complete excision with ample uninvolved margin is documented. In 1988, Daya and Scully reported four subjects that remained disease-free despite being treated by cervicectomy or polypectomy, without any adjuvant treatment.

Irrespective of the chemotherapy chosen, our experience shows that a conservative approach may be reasonable after complete removal of a polypoid sarcoma botryoides arising from the cervix. Updated follow-up techniques such as hysteroscopy, colposcopy and transvaginal ultrasound may be useful for early detection of recurrences and do not require admission to the hospital. Hysterectomy may represent the definitive treatment in case of recurrence but our first case shows that small polypoid recurrences may still be managed successfully without hysterectomy.

The risk of metastatic spread remains a matter of some concern;however, we did not find any report of metastatic disease among the early cases treated conservatively in the last decade. Some authors (Perrone et al, 1990) described a woman with local

© Cancer Research Campaign 1999

and distant recurrence after radical hysterectomy for a sarcoma botryoides with deep infiltration into the cervical stroma. Since one of our patients had local recurrence despite receiving chemotherapy, the effectiveness of this chemotherapy regimen in sterilizing distant micrometastases remains debatable.

In conclusion, conservative treatment of cervical polypoid sarcoma botryoides is possible. The use of updated follow-up techniques allows for early detection of persistent/recurrent tumour. The use of adjuvant chemotherapy deserves further investigation.

### REFERENCES

Brand E, Berek JS, Nieberg RK and Hacker NF (1987) Rhabdomyosarcoma of the uterine cervix: sarcoma botryoides. *Cancer* **60**: 1552–1556

Daniel WW, Koss LG and Brunschwig A (1959) Sarcoma botryoides of the vagina. *Cancer* **12**: 74–76

Daya DA and Scully RE (1988) Sarcoma botryoides of the uterine cervix in young women: a clinicopathological study of 13 cases. *Gynecol Oncol* **29**: 290–304

Ghavimi F, Exelby PR and D'Angio GJ (1975) Multidisciplinary treatment of embryonal rhabdomyosarcoma in children. *Cancer* **35**: 677–686

Gordon AN and Montag TW (1990) Sarcoma botryoides of the cervix: excision followed by adjuvant chemotherapy for preservation of reproductive function. *Gynecol Oncol* **36**: 119–124

Hays DM, Shimada H, Raney RB, Teffet M, Newton W, Crist WM, Lawrence W Jr, Ragab A and Maurer HM (1985) Sarcoma of the vagina and uterus: The Intergroup Rhabdomyosarcoma Study. *J Pediatr Surg* **20**: 718–724

Hilgers RD (1975) Pelvic exenteration for vaginal embryonal rhabdomyosarcoma. A review. *Obstet Gynecol* **45**: 175–180

Holton CP, Chapman KE and Lackey RW (1973) Extended combination therapy of childhood rhabdomyosarcoma. *Cancer* **32**: 1310–1316

Leyvraz S, Bacchi M, Cerny T, Lissoni A, Sessa C and Herrmann R (1995) A phase I trial of intensification of ifosfamide and adriamycin with GM CSF for the treatment of sarcomas. *Proc Am Soc Clin Oncol* **14**: A 1689

Lin J, Lam SK and Cheung TH (1995) Sarcoma botryoides of the cervix treated with limited surgery and chemotherapy to preserve fertility. *Gynecol Oncol* **58**: 270–273

Montag TW, D'Ablaing G, Schlaerth JB, Gaddis O Jr and Morrow CP (1986) Embryonal rhabdomyosarcoma of the uterine corpus and cervix. *Gynecol Oncol* **25**: 171–194

Ortega JA (1979) A therapeutic approach to childhood pelvic rhabdomyosarcoma without pelvic exenteration. *J Pediat* **94**: 205–209

Perrone T, Carson LF and Dehner LP (1990) Rhabdomyosarcoma with heterologous cartilage of the uterine cervix. A clinicopathologic and immunohistochemical study of an aggressive neoplasm in a young female. *Med Pediatr Oncol* **18**: 72–76

Rutledge F and Sullivan M (1967) Sarcoma botryoides. *Ann NY Acad Sci* **142**: 694–708

Zeisler H, Mayerhofer E, Joura A, Bancher Todesca D, Kainz C, Breitenenecker G and Reinthalier A (1998) Embryonal rhabdomyosarcoma of the uterine cervix. Case report and review of the literature. *Gynecol Oncol* **69**: 78–83

*© Cancer Research Campaign 1999*
